# Non-Viral Delivery of CRISPR/Cas Cargo to the Retina Using Nanoparticles: Current Possibilities, Challenges, and Limitations

**DOI:** 10.3390/pharmaceutics14091842

**Published:** 2022-09-01

**Authors:** Ahmed Salman, Ariel Kantor, Michelle E. McClements, Gemma Marfany, Sonia Trigueros, Robert E. MacLaren

**Affiliations:** 1 Nuffield Department of Clinical Neurosciences, University of Oxford, Oxford OX3 9DU, UK; 2 Department of Genetics Microbiology and Statistics, University of Barcelona, 08007 Barcelona, Spain; 3 CIBERER, University of Barcelona, 08007 Barcelona, Spain; 4 Department of Zoology, University of Oxford, Oxford OX1 3SZ, UK; 5 Oxford Eye Hospital, Oxford OX3 9DU, UK

**Keywords:** CRISPR, gene therapy, retina, non-viral delivery, nanoparticles, AAV

## Abstract

The discovery of the CRISPR/Cas system and its development into a powerful genome engineering tool have revolutionized the field of molecular biology and generated excitement for its potential to treat a wide range of human diseases. As a gene therapy target, the retina offers many advantages over other tissues because of its surgical accessibility and relative immunity privilege due to its blood–retinal barrier. These features explain the large advances made in ocular gene therapy over the past decade, including the first in vivo clinical trial using CRISPR gene-editing reagents. Although viral vector-mediated therapeutic approaches have been successful, they have several shortcomings, including packaging constraints, pre-existing anti-capsid immunity and vector-induced immunogenicity, therapeutic potency and persistence, and potential genotoxicity. The use of nanomaterials in the delivery of therapeutic agents has revolutionized the way genetic materials are delivered to cells, tissues, and organs, and presents an appealing alternative to bypass the limitations of viral delivery systems. In this review, we explore the potential use of non-viral vectors as tools for gene therapy, exploring the latest advancements in nanotechnology in medicine and focusing on the nanoparticle-mediated delivery of CRIPSR genetic cargo to the retina.

## 1. Introduction

**Figure 1:**
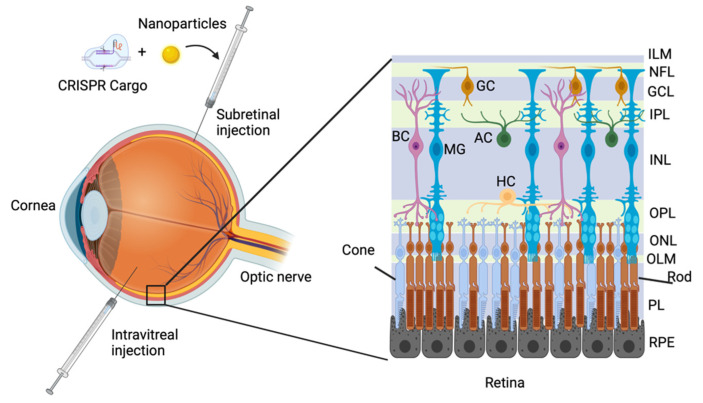
Delivery routes of CRISPR/Cas cargo to the mammalian retina. A representative illustration of the major delivery routes of CRISPR/Cas cargo to the retina represented by subretinal and intravitreal injections. The mammalian retina is divided into three main laminar layers: the outer nuclear layer (ONL), the inner nuclear layer (INL), and the ganglion cell layer (GCL). There are six different retinal neuronal cell types and one glial cell type distributed within the nuclear layers: the rod and cone photoreceptor nuclei are located in the ONL, whereas the nuclei of the bipolar (BC), horizontal (HC), amacrine (AC), and Müller (MG) cells are located in the INL. The cell bodies of the ganglion cells (GC) are located in the GCL. The processes of the different cells are extended into two plexiform layers. Processes from the photoreceptor cells are extended into the outer plexiform layer (OPL) to form synapses with the retinal neurons. Processes from the bipolar, horizontal, amacrine, as well as Müller cells are extended into the inner plexiform layer (IPL). The axons of the ganglion cells are directed into the optic nerve through the nerve fibre layer (NFL).

Inherited retinal diseases, also known as retinal dystrophies, represent a diverse group of conditions that can cause debilitating vision or eventually lead to blindness [1]. The pathway of light stimulus through the retina starts in the outer retina, with the stimulation of photoreceptors—rods and cones—by a photon of light. Upon this stimulation, a circuitry of light conductivity transmits the light signal from the outer to the inner retina, where axons of the ganglion cell layer leave the retina through the optic nerve and connect with the visual centres in the brain (Figure 1). Unlike cone photoreceptors, who can respond to a broad range of light wavelengths—making them important for daytime and night vision,—rods are highly sensitive and can respond to single photons, thus setting an absolute visual threshold [2,3]. Rod photoreceptors make synapses with the ganglion cells through distinct classes of rod bipolar cells that are conserved across vertebrate species [4,5,6]. In the mammalian retina, rod photoreceptors establish synapses using the excitatory neurotransmitter glutamate with a distinct class of bipolar cells called the ON-bipolar cells (ON-BCs) [7,8], who in turn synapse to another set of intermediate neurons, the amacrine cells. Light signals that are converted into chemical and electrical signals from rod photoreceptors finally reach the ganglion cells via synapses formed between the latter and amacrine cells.

Several reasons make the retina an exceptional model for developing treatments for genetic diseases, such as its easy surgical access for delivery of therapeutic agents via intraocular injections. Moreover, the retina, similar to the brain, has a relatively immune privileged blood–retinal barrier [9], which reduces the risk of a generalized immune response to the therapy and makes safety concerns local to the eye. In addition, it is easy to evaluate the efficacy of a given therapy due to the availability of several techniques, such as scanning laser ophthalmoscopy (SLO) and electroretinography (ERG), which are well-established tests to evaluate retinal structure and function. Standard behavioural tests also exist to assess the processing of visual inputs, such as behavioural light avoidance (BLA) and optomotor response (OMR).

The current therapeutic approaches to treat inherited retinal disease include cell- and nucleic acid-based therapies, which aim to replenish a specific cell type population in the retina or to restore the molecular function of a mutated gene by either gene supplementation or splicing correction, respectively. Adeno-associated virus (AAV)-based gene therapy approaches for retinal diseases have been well established in the last two decades with dozens of ongoing trials, being the therapy used to treat *RPE65*-associated Leber congenital amaurosis, which is the first FDA-approved and commercialized gene therapy that is directly administered to target a disease caused by mutations in a specific gene [10]. However, gene supplementation strategies are limited to loss-of-function mutations, and many genes are precluded due to the packaging constrains of AAV. Thus, for large genes or dominant disorders, alternative strategies are required. The clustered regularly interspaced short palindromic repeats (CRISPR)-Cas system provides a potential solution to the shortcomings and limitations of gene supplementation due to its ability to target, bind, and repair DNA [11,12,13]. The CRISPR/Cas system is capable of mutation-specific targeting irrespective of the pattern of inheritance, which means treatment of disease that is not amenable to gene supplementation is now possible. Indeed, the first FDA-approved clinical trial for in-body CRISPR gene therapy was a treatment for inherited retinal disease by using AAV-derived vectors to target the deep-intronic c2991 + 1655A > G single nucleotide mutation in the *CEP290* gene [14]. However, the big molecular size of the CRISPR constructs is also problematic as it hampers their packaging in AAV particles, and an alternative vehicle to deliver the CRISPR/Cas cargo might be necessary.

Non-viral gene delivery has recently been considered an alternative and promising method for gene therapy, where the hurdles of packaging size limitation and safety can potentially be overcome. Non-viral gene delivery platforms can be divided into physical and chemical methods [15]. The physical methods take advantage of physical phenomena that disrupt the plasma membrane of a cell to gain access to the cytoplasm, and subsequently, the nucleus. These methods vary from classic gene delivery techniques, such as needle injections, which in the retina can be subretinal or intravitreal [16], to particle bombardment or a gene gun using pressurized helium gas against the plasma membrane [17,18]. Electroporation uses short, high-voltage pulses to create transient pores in the plasma membrane [19,20], whereas sonoporation generates the pores through the application of low-frequency- [21] or high-intensity-focused [22] ultrasound irradiation.

The chemical methods for non-viral gene delivery can be broadly divided into organic and inorganic strategies depending on the nature of the packaging molecules [15]. Organic methods mainly refer to either cationic lipids or polymers, such as lipid nanoparticles and poly-etheylenimine (PEI), respectively, which are positively-charged and therefore interact with negatively-charged DNA. Inorganic methods are a rapidly expanding gene delivery toolkit due to their structural and physical properties, which can be adaptable to a variety of cell and tissue types [23]. Depending on the material each with specific optical, physical, electrical, or magnetic properties—inorganic methods can include gold nanoparticles, silica nanoparticles, carbon nanotubes, quantum dots, and magnetic nanoparticles [24].

In this review, we will explore the delivery of CRISPR cargo to the eye using non-viral gene delivery techniques. The suitability of different delivery methods will be discussed, elaborating on some of the challenges that the eye, particularly the retina, might present when considering non-viral gene delivery platforms. The advantages, disadvantages, and limitations of different delivery techniques will also be discussed.

## 2. CRISPR Therapeutic Mechanisms

The recent discovery of the mechanisms of CRISPR/Cas9 in the bacterial immune system, and the subsequent adaptation into a powerful gene editing tool, has revolutionized the field of molecular biology and generated excitement for the potential of novel therapeutic approaches to treat human conditions [25]. The CRISPR/Cas system encompasses a variety of components that differ widely in mechanisms of action and offer therapeutic potential by direct genome interaction and/or editing. CRISPR-mediated genome editing involves the generation of a Cas9-induced DNA double-strand break, which is repaired by the cell using either non-homologous end joining (NHEJ) mechanisms or homology-directed repair (HDR) [26]. Although HDR-mediated gene editing can be harnessed to insert a specific DNA template for the precise restoration of the DNA sequence, this pathway is characterized by low efficiency and high rates of undesired indel mutations that hamper the potential benefit from repairing the mutation [27].

The development of CRISPR/Cas-mediated single base pair editing systems (or ‘base editing’ systems) have a remarkable potential as therapeutic tools to correct disease-causing mutations in the human genome [28,29,30]. Whereas HDR repair pathways are restricted to the S and G2 phases of the cell cycle, base editing employs cellular mismatch repair machinery and can be applied to reverse genetic defects in both dividing and terminally-differentiated cell types. Gene therapy is a major area where DNA base editing toolkits can be applied because they have already been adapted to characterize, model, and correct the underlying causes of human genetic conditions. The development of targeted cytosine and adenine base editors enables the targeted correction of all four transition mutations, and recent engineering of base editor architectures have expanded the range of DNA editing to transversion mutations and may allow for targeting of more complex compound edits [29]. Moreover, recent developments in site-directed RNA editing provide a novel base editing approach that targets the transcriptome rather than the genome.

In addition to genome editing, CRISPR/Cas9 can be used for transcriptional regulation, in which catalytically-inactivated “dead” Cas9 (dCas9) is fused to transcriptional effectors to repress genes directly (CRISPRi) or modified to act as a functional transcriptional activator (CRISPRa). Epigenetic repression provides a safer alternative to indel-mediated gene disruption, whereas CRISPRa may have utility for the treatment of haploinsufficiency conditions, in which one copy of the gene is not sufficient to assure a normal phenotype [31].

Finally, prime editors, the latest addition to the CRISPR genome-engineering toolkit, expands the scope of donor-free precise DNA editing to not only all transition and transversion mutations, but insertion and deletion mutations. The most recent CRISPR technologies, including PASTE [32] and TwinPE [33], build on the PE system to install large genetic insertions. Collectively, the CRISPR toolkit has remarkable potential as a therapeutic tool to correct disease-causing mutations in the human genome [13].

**Figure 2:**
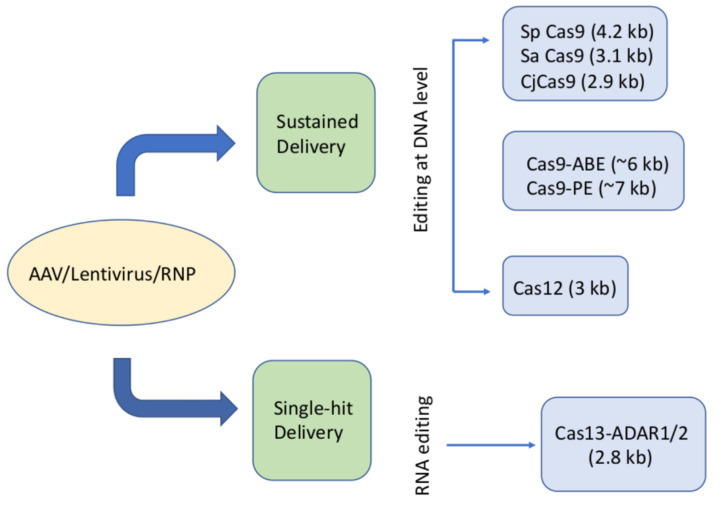
Different Cas-related proteins and their main characteristics for potential use in CRISPR-based gene therapies. A schematic diagram showing the different classes of Cas proteins used to target/edit retinal cells with their relative sizes. The current delivery platforms used with the CRISPR/Cas cargo are AAV, lentivirus, and ribonucleoproteins (RNPs). CRISPR editing can be achieved either in a sustained delivery acting on DNA or as a single-hit even acting on RNA.

Despite tremendous potential in genome engineering for the treatment of inherited retinal diseases, significant challenges remain with the delivery of CRISPR reagents into retinal cells, and most editing systems are well beyond the packaging constraints of a single AAV vector. Thus, the development of alternative delivery approaches is critical for unlocking the great therapeutic potential of these systems. Therefore, one of the most interesting fields in CRISPR-technology is the identification and development of Cas-related proteins from other bacterial organisms. As stated, the large size of Cas9 hinders the packaging of all the CRISPR-components into AAVs, whereas several other alternatives, with different sizes and properties (e.g., targeting of DNA or RNA molecules), provide an ample field of diversification tools (Figure 2).

## 3. Current Delivery of CRISPR Reagents

**Figure 3:**
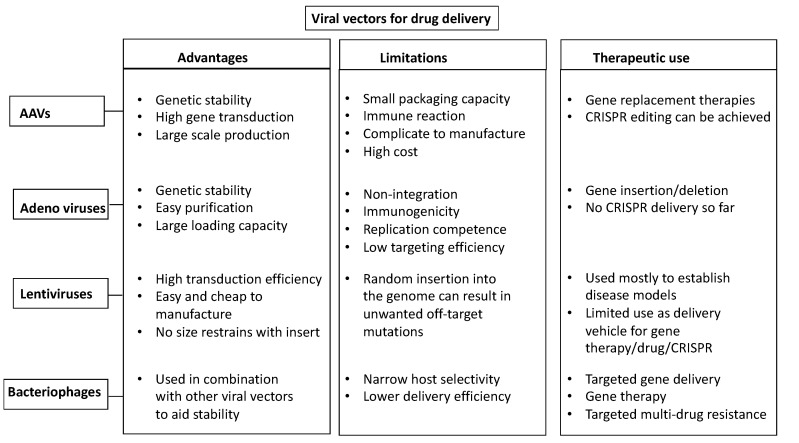
Viral-based delivery vectors. Schematic diagram showing the advantages, limitations, and therapeutic uses of the four main viral-based vehicles for CRISPR-reagents delivery: AAVs, Adenoviruses, Lentiviruses, and Bacteriophages.

Classical genome engineering methodologies such as zinc finger nucleases (ZFNs) and transcription activator-like effector nucleases (TALENs) have limited therapeutic advantages due to their low in vivo efficacy [26] and their ability to only achieve genome editing ex vivo. The advantage and novelty of the CRISPR/Cas9 system is its versatility, as it is capable of editing in vitro, ex vivo, and in vivo [34,35]. Proof-of-principle editing of the CRISPR/Cas system as well as targeting of common mutations in retinal disease has already been achieved [36,37,38,39]. The potential difficulty of delivering any therapeutic agent is a hurdle, however, this obstacle has been overcome in the retina by AAV-based delivery, one of the four classic viral drug delivery vehicles known (Figure 3). AAVs are genetically stable, relatively safe to handle, have a minimal toxicity to host cells, have high transduction efficiency, and elicit a minimal immune response in the retina [40]. This was evident by the approval of the first CRISPR in vivo genome engineering clinical trial for inherited retinal disease by editing the *CEP290* gene in Leber congenital amaurosis [14]. The potential of AAVs in targeting the photoreceptors has already been investigated for some time now, along with the limitations involved. The relatively small packaging capacity of AAV, accompanied by the limited type of inherited retinal dystrophies in which gene supplementation is a potential therapeutic approach (recessive/X-linked), underscore the need to explore alternative delivery platforms to overcome such limitations. Even when considering therapeutic strategies based on the CRISPR/Cas system, where the CRISPR/Cas cargo can be packaged as an all-in-one vector approach, which makes the delivery process more straightforward, the limited packaging capacity of AAV is still a relevant issue. Moreover, the prolonged expression of viral vectors represents a major concern in achieving a safe therapy with such a highly-efficient genome editing system as the CRISPR/Cas system.

## 4. Nanomaterials for CRISPR Reagents Delivery Systems

Several types of nanostructures have already been developed, and their in vivo capability for CRISPR reagent delivery has been tested. Even though nanodelivery systems are still in their infancy, several nanocarriers, including polymers, lipids, porous silicon, mesoporous silica nanoparticles, and metal-organic composites, are being developed for gene delivery due to their low immunogenicity, high biocompatibility, and superior cargo delivery capabilities.

The clinical development of nanoparticles (NPs) has expanded into a broad range of diagnostic and therapeutic applications in recent years. Nanoparticles are designed to overcome current therapeutic limitations, such as crossing systemic barriers and toxicity. The different classes of nanoparticles (e.g., lipid based, polymeric, or inorganic) are designed to optimize delivery platforms in a personalized manner to enter the era of precision medicine [41,42]. NPs have the potential to improve the solubility and stability of encapsulated cargos (such as the CRISPR/Cas cargo), promote safe transport across biological membranes, and prolong the circulation and expression of the therapeutic reagent in a tissue-specific manner to increase safety and efficacy [43,44,45].

## 5. Nanostructures Currently Used in Delivery Systems

### 5.1. Lipid-Based NPs

Lipid-based nanoparticles are typically spherical and comprise at least one lipid bilayer surrounding one internal aqueous compartment [46]. Lipid-based NPs can be subdivided into liposomes and lipid nanoparticles (LNPs). Liposomes are commonly used in biomedicine, having many clinical uses [47,48,49], including liposomal verteporfin for treatment of macular degeneration [50]. Their flexible biophysical properties allow them to carry hydrophilic, hydrophobic, and lipophilic drugs. Lipid NP vesicles can be altered during synthesis to modify their size, surface charge, lipid composition, and surface modifiers (ligands and polymers), making them very potent drug delivery vehicles [51,52,53,54].

LNPs are commonly used in nucleic acid delivery and differ from liposomes in their micellar structure and spherical form in aqueous solutions [55]. LNPs have four major components: cationic or ionizable lipids that complex with negatively-charged nucleic acids; phospholipids, which form the main particle’s structure; cholesterol, which aids stability and membrane fusion; and PEGylated lipids, which further improve stability and circulation [56,57]. Due to their efficient and manipulated structure, LNPs have been considered as vehicles for the CRISPR/Cas cargo [58]. However, the difficulty in controlling their size, uniformity, and stability in vivo severely affects their clinical applications.

Researchers have since focused on other methods to maintain the integrity of the CRISPR ribonucleoprotein (RNP) by adding permanent modulatory lipid components. These modulations mediate the encapsulation of the RNPs and help to improve editing efficiency in muscles, the brain, liver, and lungs in vivo [59].

### 5.2. Polymeric NPs

Polymeric NPs have a variety of structures and characteristics, as they are synthesized from monomers and polymers of natural or synthetic materials, thereby enabling precise control of multiple NPs feature. Therefore, they are generally good drug delivery vehicles as they are biocompatible and simple [46]. Polymeric NPs have various drug delivery capabilities, as therapeutics can be encapsulated within the NP core, either being chemically conjugated to the polymer or bound to the NP surface. This enables delivery of hydrophilic and hydrophobic macromolecules as well as proteins [60,61,62,63,64]; hence, they are potentially ideal CRISPR/Cas cargo carriers.

Polymeric NPs include many types, from co-polymerised (e.g., poly(ethylene glycol) (PEG) and poly(dimethylsiloxane) (PDMS)) to charged polymers (e.g., poly(ethylamine) (PEI) and poly(amidoamines) (PAMAM)) [46], all are prepared with varying methods that confer different delivery capabilities.

Overall, polymeric NPs are very good delivery candidates for CRISPR/Cas cargo delivery because they are biodegradable, water soluble, biocompatible, stable during storage, and their surface is easy to manipulate to achieve target delivery, thus allowing them to deliver proteins and genetic material to specific cells or tissues, making them an ideal delivery vehicle for gene therapy and diagnostic purposes. However, as with many drug delivery vehicles, there is a disadvantage associated with their use due to the increased risk of particle aggregation, which leads to toxicity. Although many polymeric nanocarriers are currently undergoing testing in numerous clinical trials, only a limited number of polymeric nanomedicines are currently approved by the FDA [49].

### 5.3. Inorganic NPs

Inorganic NPs have unique physical, optical, magnetic, and electrical properties, making them a very promising prospect for the delivery of therapeutic and diagnostic applications. Inorganic NPs are made of inorganic materials such as gold, silica, and iron, and they are formulated to have a variety of sizes, structures, and geometries. Gold nanoparticles (AuNPs) are used in various forms, such as nanospheres, nanorods, nanoshells, and nanocages [65]. The properties of the base material of the inorganic NPs determine their physicochemical properties, and therefore, their potential use. For example, AuNPs of a particular shape and size possess free electrons on their surface area in a continuous oscillation state at a given frequency, conferring their photothermal properties [66]. AuNPs are very promising for CRISPR/Cas cargo delivery due to their flexible properties and ability to penetrate the plasma membrane without disturbing the phospholipid bilayer. They are very small and easy to formulate, granting them additional delivery capabilities [65].

The plasmonic properties of the AuNPs make them, in theory, a very promising non-viral delivery platform for CRISPR/Cas cargo: they allow flexibility in cargo packaging and can be manipulated to be tissue- and cell-type specific. Given their light absorbance properties, they allow temporal activation upon need, which can be very advantageous to reduce off-target effects. Although AuNPs are non-toxic, the toxicity of other nanomaterials is still under examination. Therefore, toxicity concerns over inorganic NP formulation and potential in vivo accumulation of heavy metals still need to be addressed.

Another commonly used inorganic NPs are magnetic iron oxide NPs, which are composed of magnetite (Fe_3_O_4_) or maghemite (Fe_2_O_3_). Magnetic iron oxide NPs possess superparamagnetic properties and have shown potential in delivering thermal-based therapeutics [67].

Mesoporous silica nanoparticles (MSNs) are nanoparticles made of silica with pores of about 2- to 50-nm that endorse them with unique physicochemical properties. These nanocarriers can be prepared in a variety of sizes and shapes, including nanohelices, nanotubes, nanozigzags, and nanoribbons. The ability to tailor pore sizes, volumes, and surface area—as well as easy encapsulation of drugs, proteins, and biogenetic materials—makes MSNs a versatile delivery vehicle. Given these advantages, MSNs can be used to carry both small and large molecules. Even though MSNs are hardly degraded in the body, they seem to be biocompatible and exhibit low toxicity. MSNs are promising delivery systems that can be used for: (I) improving drug solubility, (II) selective targeting for targeted therapy, and (III) controlled dosage and smart behaviour (internal and external stimuli-responsive drug delivery) [68,69].

MSNs have been widely used to load small molecule chemotherapy drugs, nucleic acids, proteins, and other biological macromolecules. They have been successfully applied in basic research of tumour multimodal treatments [70]. Given that they are inexpensive, are capable of large-scale synthesis, have the potential for surface functionalization, and have high biocompatibility, finding new MSNs suitable for cargo delivery is becoming a priority for nanodelivery applications [71].

**Figure 4:**
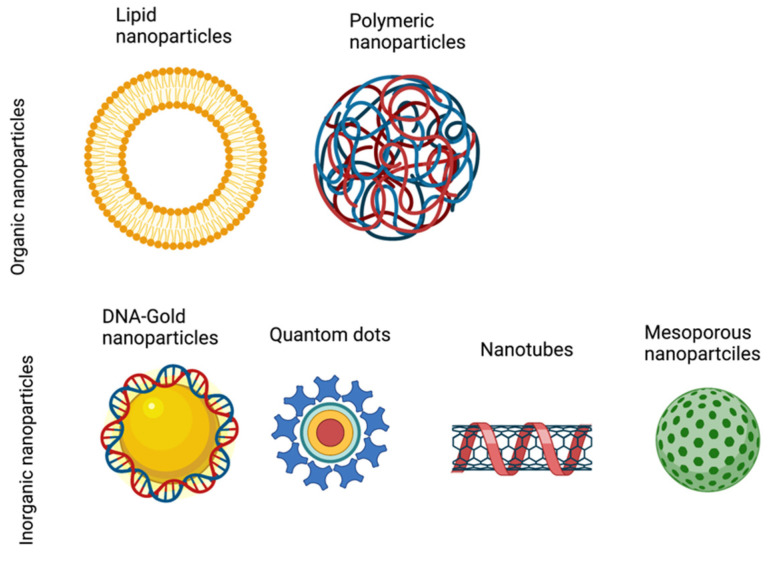
Types of nanoparticles. Schematic diagram showing the different types of nanoparticles, which can be classified into two categories: Organic nanoparticles, including lipid and polymeric particles; and inorganic nanoparticles, including gold nanoparticles, quantom dots, nanotubes, and mesoporous nanoparticles.

As summarized in this review (Figure 4), inorganic nanoparticles have multiple therapeutic applications. Calcium phosphate and mesoporous silica NPs have both been used in drug and gene delivery [72,73], whereas quantum dots, which are typically made of silica, have been used primarily in medical imaging applications, with promise shown in in vivo diagnostics too [74,75].

## 6. Hurdles to Overcome for CRISPR Nanoparticle Delivery to the Retina

The in vitro and in vivo delivery of therapeutic agents to the retina remains challenging for five main reasons: (i) unwanted off-target effects; (ii) activation of the immune response; (iii) toxicity to cells; (iv) difficulty in reaching target cells; and (v) rapid degradation of the therapeutic agent that renders it ineffective, or in some cases, just the contrary, not being degraded fast enough before starting to pose a safety concern [76,77]. Those challenges exist when targeting any cell or tissue type. The retina has a unique anatomical structure that provides protection (immune privilege), as it is enclosed by the choroid and retinal pigment epithelium. It is made of multiple layers of cells that collaboratively work together to transduce light/electric signals to process vision [5]. Some of those cells have a unique anatomy that makes the delivery of nucleic acids and proteins challenging, such as photoreceptors. The photoreceptors’ outer segment is particularly challenging for the targeted delivery of therapeutic agents due to the highly-specialized structural arrangement of the plasma membrane, which is made of multiple stacks of phospholipid bilayers—which are hard to penetrate. The successful delivery of CRISPR/Cas cargo to the retina must combine safe delivery and effective gene editing; to achieve this, there are a number of barriers to overcome.

### 6.1. Physical Barriers (The Retinal Pigment Epithelium/Bruch Membrane and the Choroid)

The retinal pigment epithelium (RPE) is a monolayer of pigmented cells that provide support to the adjacent photoreceptors. The RPE is intimately interconnected to photoreceptors, and vision is thus highly dependent on the connection between these two cell layers [78]. The RPE supports photoreceptors by recycling visual cycle components, clearance of the photoreceptor membrane, transport of nutrients, and clearance of waste products generated during daily photoreceptors renewal [79]. The RPE is part of the outer blood–retina barrier (BRB) along with the Bruch membrane (BM), which is a highly-organized layer of basement membrane situated between the RPE and the choroid—a thin layer of tissue made up almost entirely of blood vessels. One of the hallmarks of RPE maturation, which is essential for its function, is the formation of tight junctions between the RPE cells. The three stages of tight junction development have been described [80,81,82,83,84,85,86]. The formation of tight junctions establishes the BRB and regulates the epithelial transport carried out by the RPE. Systemic drug or nucleic acid delivery to the eye is more challenging than to other organs due to the multiple ocular barriers, including the BRB, which is meant to anatomically and physiologically protect the eye from toxins. Drug delivery to the eye includes three major routes: anterior segment, posterior segment, and intravenous delivery [87,88]. Effective drug delivery for inherited retinal dystrophies requires posterior segment delivery. For the delivery of therapeutic agents, including CRISPR/Cas components, penetrating the impermeable BRB represents a significant hurdle, but this can be overcome through a subretinal injection between the RPE and the photoreceptor outer segment using a microneedle. Subretinal injection is a well-established technique that is highly successful at targeting cells in the subretinal space, such as the photoreceptors [89,90], and thus provides an ideal delivery system for the delivery of the CRISPR/Cas components into the subretinal space. Nonetheless, subretinal injection is a rather aggressive surgical intervention, as the introduction of the solution volume forces photoreceptors apart from the RPE, which might cause a similar effect to retinal detachment. Currently, subretinal injections are only considered for one shot delivery. Another route to deliver CRISPR/Cas cargo to the retina is via intravitreal injection [16], which is less aggressive and can be repeated periodically. Intravitreal injections are ideal for the treatment of conditions with blood vasculature abnormalities, such as AMD and retinal diabetic macular oedema, since the inner blood vessels are situated on the vitreous side in the inner retina. The downside of intravitreal injection is that it carries a risk of inflammation [91]. There is also a less invasive route of drug delivery, which is the topical application of the CRISPR cargo. However, topical delivery can be hampered by hurdles, such as tear formation (the short contact time of the drug to the eye surface before tear formation can clear away the drug); the lipophilic membranes (cornea and conjunctiva), which are hard to permeabilize [92]; and the physical distance to the retina. Overall, the highest limitation is penetrance: it is difficult to envision how the topical delivery of the CRISPR cargo can ensure uptake by retinal cells.

### 6.2. Photoreceptor Outer Segment

Intracellular-targeted drug delivery requires the therapeutic agent to first reach the surface of the target cells. Retinitis pigmentosa (RP), which describes a group of genetically heterogeneous rod-cone dystrophies, is the most common form of inherited retinal degeneration with a prevalence of 1 in 4000 and nearly 2 million affected individuals worldwide [93,94]. RP is characterized by the progressive loss of first rod, and later, cone photoreceptors, leading to progressive loss of vision. Specialized invasive delivery methods, like subretinal injections, can deliver the therapeutic agents to the surface of the photoreceptors’ outer segment. The outer segment of each rod photoreceptor consists of hundreds of numerous flattened, stacked membrane discs that contain rhodopsin and other proteins relevant for phototransduction (for a full review on the structure of the outer segment, see Fletcher et al., 2011 [95]). The discs are composed of double phospholipid bilayers that give the selectivity function of the plasma membrane (hydrophobic on the outside and hydrophilic from the inside). The presence of cholesterol, an essential membrane constituent, reduces the permeability of the plasma through interaction with the phospholipids, which results in the thickening of the bilayer [96], making it hard for any therapeutic agent to penetrate.

The first successful in vivo targeting of the photoreceptors, as well as the RPE, was achieved using AAV serotype 2 in rodents in 1996 [97,98]. So far, recombinant AAV2 is the standard successful gene therapy tool used in numerous clinical trials for the treatment of inherited retinal dystrophies, such as Leber congenital amaurosis [99,100,101,102,103,104], retinitis pigmentosa [105,106], and choroideremia [107], due to its ability to transduce post-mitotic neurons as well as proliferating cells (e.g., photoreceptors and RPE). The ability of AAV to diffuse through the multiple membranous discs of the photoreceptor outer segment makes it a very efficient gene delivery tool [108,109].

The outer segment is in a constant state of degradation and renewal, as part of the visual signal transduction cascade. The membrane discs are continuously displaced to the apical tip of the outer segment as new discs are formed, and the old discs are shed and phagocytosed by the RPE [110]. In want of more conclusive data, this continuous renewal/degradation process might as well represent a therapeutic challenge for CRISPR/Cas cargo delivery, as the cargo might be degraded or phagocytosed out of the outer segment before reaching the photoreceptor nucleus.

### 6.3. Physiological Barriers (Osmolarity/pH)

It is important to consider the physiochemical properties of drugs in in vivo delivery to any tissue type. For the CRISPR/Cas cargo, there are a number of important properties that need to be considered carefully, such as the hydrophilicity, large molecular weight, and metabolic instability of the CRISPR RNPs in a physiological environment (e.g., the cytoplasm) [111]. The low stability and short half-life of the CRISPR/Cas RNPs at either physiological pH and temperature, or during formulation/storage, pose a significant burden for any in vivo drug delivery platform. Most of the therapeutic proteins, including the CRISPR/Cas cargo, are hydrophilic. The lipophilic nature of the plasma membrane restricts the spontaneous diffusion or passive absorption of hydrophilic molecules, hence the active transport of macromolecules across the membrane might be necessary. One of the common routes for the entry of proteins and peptides from the extracellular space into the cytoplasm is mediated by active transport by receptor-mediated endocytosis [112], a process that generally occurs by forming endosomes that enclose the proteins/peptides. When in the cytoplasm, the endosomes eventually mature into or fuse with lysosomes so that most of the internal cargo gets degraded by lysosomal enzymes. Therefore, the endosomal entry of the CRISPR/Cas reagents might not represent a very promising strategy. To bypass endocytosis, mechanical delivery routes, such as microinjections and electroporation, have been used to deliver drugs across the plasma membrane. However, those routes are often inefficient, invasive, and require specialized surgical equipment.

Another major challenge in protein/peptide-based drug delivery to the eye is the epithelial tight junctions between the RPE cells. The molecular weight of most therapeutic proteins is >1000 Da [113], and the molecular weight of *Streptococcus pyogenes* Cas9 (*SpCas9*) is approximately 160 KDa in size [114]. The human retina limits the diffusion of molecules greater than 76 KDa due to the thickness of the inner and outer plexiform layers, and any macromolecules exceeding 160 KDa often fail to reach the inner retina by diffusion [115].

Therapeutic reagents such as the CRISPR/Cas cargo may encounter various intracellular physical and chemical degradation pathways upon penetration of the plasma membrane. Proteins have complex secondary, tertiary, and quaternary structures, which may hinder their biological stability. Physical pathways that are involved in protein instability include denaturation, absorption, aggregation, and precipitation, with chemical pathways including deamidation, oxidation, reduction, proteolysis, disulphide exchange, and beta-elimination [116]. Moreover, the conformational transformation of proteins to their inactive form in physiological pH, temperature, and high salt concentration may all contribute to the instability of therapeutic reagents [117]—although this type of potential barrier to the CRISPR/Cas cargo is not unique to the retinal cells and is shared with other cell types in the body.

### 6.4. Technical Barriers

There are ethical concerns with the therapeutic use of the CRISPR/Cas9 system due to the cultural and social understanding and acceptance of gene editing. However, there are many other concerns, for instance, safety concerns that must be addressed first to determine its viability as a therapeutic reagent. CRISPR/Cas9-based gene editing in animal zygotes is now commonly used in the generation of transgenic models [118,119,120,121]. However, concerns about the use of such a technology for gene editing in human zygotes and embryos is still a big concern in the scientific community, leading to its ban in some parts of the world [122]. Nonetheless, gene editing in somatic cells to treat a severely incapacitating disease, such as blindness, is more widely accepted.

There are several gene delivery methodologies to the eye/retina/RPE that have been reported in the literature with variable degrees of success. The cheapest and easiest gene therapy constructs are plasmids, as they are easy to propagate and purify in large quantities without the need for specialized equipment. It is relatively easy to design a plasmid that contains the different CRISPR/Cas9 system components in an all-in-one plasmid, including the guide RNA, or it can be delivered in a dual transfection strategy by delivering the Cas9 and the guide RNA separately. Plasmids normally contain bacterial replicons and selectable marker sequences that can be removed to make what are called “DNA-minicircles”, which have the advantage of containing only the DNA of interest to be delivered into the target cells and eliminate safety concerns regarding bacterial components of the plasmid while preserving the highly-active transcription of the therapeutic gene [123]. The main challenge of delivering plasmids into cells in vivo is that it requires creating pores in the plasma membrane as a route of entry. Plasmids and minicircles can be delivered to the vitreous or subretinal space by intravitreal or subretinal injections with various physical methods to aid its entry in the cytosol. Those methods include the microbubble disruption of the plasma membrane or electroporation to create temporary pores [124,125,126,127,128,129] and have been used to deliver large amounts of plasmids to the retina to transfect different cell types. The disadvantage of such approaches is that the disruption of the lipid bilayer structure causes the death of a significant number of targeted cells.

Another promising methodology for CRISPR delivery to the eye is the use of nanoparticles containing the CRISPR/Cas9 components. Inorganic nanomaterials, such as gold nanoparticles, magnetic nanoparticles, carbon nanotubes, and quantum dots, are emerging therapeutic tools that carry unique structural and physical properties that can be tailored to suit therapeutic needs [23]. Nanomaterial therapeutic technologies have been moving at a rapid pace during the last decade. Much of the research on the applications of nanotechnologies focus on stabilizing the nanoparticles in the blood stream until they reach their target cells. Once there, nanoparticles need to cross the plasma membrane to release their cargo in the cytoplasm. However, in the retina, the direct delivery of nanoparticles to the vitreous or the subretinal space by intravitreal or subretinal injections, respectively, place the nanoparticles nearby the target cells. Gold nanoparticles, for example, exhibit photothermal properties: colloidal gold exhibits localized plasmon surface resonance (LPSR) because gold nanoparticles can absorb light at a specific wavelength [130]. The photons absorbed from light generate an electrostatic gradient across the phospholipid bilayer. This transfer in the status of electron energy across the nanoparticles and the plasma membrane creates small temporary pores in the phospholipid bilayer that are large enough for the particles to pass through. Successful targeting of the RPE cell layer using DNA-wrapped nanoparticles has already been shown in vitro [131]. The in vivo delivery of gold nanoparticles to the retina to target specific cell types, such as the photoreceptors, represents a bigger challenge. As mentioned, the photoreceptor outer segment is packed with hundreds of membranous discs, and therefore, its membrane composition is >95% disc and <5% plasma membrane, overall consisting of 60% protein and 40% phospholipid [132].

As well as penetrating the plasma membrane of the photoreceptor outer segment, the CRISPR/Cas9 system components, whether encoded in plasmids or delivered as an RNP, must also be actively transported to the nucleus. Gene editing in adult tissue consists of targeting quiescent, non-dividing cells, and the CRISPR/Cas cargo must be imported to the nucleus to exert its effect. Current research has been focusing on manipulating the CRISPR/Cas9 system, e.g., humanizing the Cas9 sequence, introducing nuclear localization signals, and including a 72 bp SV40 enhancer sequence that is sufficient for the nuclear import of plasmids into the nucleus of non-dividing cells [133]. For CRISPR/Cas cargo delivery to the retina, we discussed packaging it into nanostructures that offer many advantages over viral-based delivery, such as lack of toxicity, avoidance of unwanted immune response to the therapeutic reagent, and the ability to target larger genes due to the flexibility with the size of DNA to be packaged. In fact, an important challenge of CRISPR homology-directed repair (HDR), one of the main strategies of gene editing using active Cas9, is the length of the DNA homology sequence to be packaged. It is possible to package DNA sequences to drive homologous recombination using the scaffolded DNA origami approach [134], which is particularly suited for packaging several kilobases of DNA into compact DNA nanostructures. Despite the promise of non-viral gene delivery, advances in effective cargo delivery to the nucleus still pose a significant barrier to achieve an effective therapy.

One of the most difficult challenges in in vivo gene editing is the efficiency of gene targeting. The major problem facing CRISPR/Cas9 editing is that differentiated target cells are quiescent. The rate of HDR and NHEJ is much higher in dividing cells than non-dividing cells [135,136,137]. Several factors determine the rate of editing with the CRISPR/Cas9 system, such as the rate of transfection/transduction and the number of the CRISPR/Cas9 components delivered to the cells, which in turn determines the rate of editing. Besides the non-viral delivery of CRISPR/Cas cargo to the retina, non-DNA-based delivery methods have also been reported, such as directly delivering the CRISPR components as pure RNPs (the guide RNA already bound to Cas9 in particles, see next section).

## 7. Vehicles beyond Viruses for CRISPR Delivery to the Retina

The non-viral delivery of the CRISPR/Cas cargo is advancing, with some clinical applications already in trials [138,139]. The pre-clinical in vivo non-viral delivery of CRIPSR/Cas9 components in several tissue and cell types have been reported [140,141,142,143]. Previous research on the potential of the non-viral delivery of nanomaterials to the retina has shown a glimpse of promise with variable degrees of success. For example, Kim and colleagues showed successful targeting and editing of the RPE cells in vivo when injecting SpCas9 with a gRNA that targets the vascular endothelial growth factor gene (VEGF) subretinally [144]. A study on CRISPR/Cas9 allele-specific targeting of the dominant S334ter mutation in the rhodopsin gene in rats was conducted by Bakondi et al. and colleagues, providing a proof-of-principle for allele-specific disruption to prevent retinal degeneration and improve visual function [145]. Huu and colleagues investigated the use of a non-invasive nanoparticle depot to release drugs for the treatment of wet AMD using a light-sensitive triggering system for cargo release from the nanoparticles [146]. On the other hand, Asteriti and colleagues investigated the possibility of delivering recombinant proteins encapsulated into lipid nanoparticles to the photoreceptors by intravitreal injections [147]. Additionally, the potential of magnetic nanoparticles as nanotools for drug release in the retina has also been explored, demonstrating the specificity and stabilization of the particles in the RPE [148].

There are numerous examples of the use of gold nanoparticles for the efficient delivery of the CRISPR/Cas9 components in vitro in the form of RNP- [149] and DNA-wrapped gold nanoparticles [131], with the latter achieved in a retinal epithelial cell line (ARPE19). The remarkable physical properties of the gold nanoparticles enable them to form an endocytic or “fusion-like” structure with the plasma membrane lipid bilayer, which aids the direct biomolecular entry to the cytoplasm without disturbing the phospholipid structure [150]. The in vivo delivery of the CRISPR/Cas cargo using gold nanoparticles has been investigated in the brain [151], muscle cells [152], and the retina (Figure 1).

The electroporation of synthetic materials, such as nucleic acid-containing nanoparticles, is potentially a potent approach for CRISPR/Cas cargo delivery to the retina. Electrotransfer and iontophoresis were developed as innovative non-viral gene-transfer treatments for ocular disease over 15 years ago [153]. Electroporation relies on the application of short pulses of relatively high-intensity electric fields to deliver DNA/RNA cargo, whereas iontophoresis is based on the application of a low-voltage electric current. At that time, the basic principle of the therapeutic potential of those techniques had been investigated and their efficient delivery of small nucleic acid fragments, such as antisense oligonucleotides, siRNA, and ribosomes, was established. Recently, their application as a non-viral gene delivery toolkit has been patented (US patent application #20210128911) to posit their potential as a game-changer in the gene therapy paradigm.

Another promising approach for CRISPR/Cas cargo delivery to the retina is the microbubble gene-gun approach. Microbubbles are currently in clinical use as mediators for ultrasound gene therapy [154,155,156]. Nanoparticle incorporation into phospholipid shells for the adaptation of clinically-approved microbubbles have been developed and established by Owen and colleagues [157], who showed that lipid vesicles containing multiple lipid-coated nanoparticles can be fragmented when sonicated, leading to the formation of microbubbles that fuse into the plasma membrane lipid bilayer. After cooling, the phospholipid membrane condenses with the nanoparticles entrapped within, thus aiding the nanoparticle penetration and delivery to the cytoplasm. It would be interesting indeed to adapt such an approach for targeting the photoreceptors, however, the question remains whether it will be enough to penetrate the multiple discs of the photoreceptors’ outer segment without disrupting the phospholipid bilayer integrity.

Recently, a new, promising non-viral gene delivery toolkit has been validated to target the retina [158]. The engineered viral-like particles (VLPs) developed by Banskota et al. and colleagues combine key advantages of viral and non-viral gene delivery toolkits. They overcome the cargo packaging limitation for CRISPR and combine the viral ability to cross the plasma membrane, without eliciting the immune response of viral vectors. VLPs are assemblies of viral proteins capable of infecting cells, but they lack the viral genetic material. Nanovesicles including the VLPs have recently emerged as a promising non-viral gene delivery vehicle for CRISPR delivery of RNPs [159,160,161,162,163,164,165]. Such an approach would be suitable for the delivery of Cas9 nucleases (active and inactive) and base editors (ABEs/CBEs); for instance, the hit-and-run approaches reported by Indikova and Indik et al. and by Lyu et al. take advantage of the one-time application of the CRISPR RNP to reduce off-target effects while achieving sustained expression of the Cas9 nuclease [162]. The nanoblade “all-in-one” homology-directed repair, or the programmed repair with modified Cas9 variants, shown by Mangeot et al. and colleagues in the embryos and liver of adult mice, represents a promising approach for the mediated transcriptional upregulation of target genes (CRISPRa) [164], whereas the exon-skipping editing in Duchenne muscular dystrophy iPSC cells shown by Gee and colleagues explores a new therapeutic avenue for mutations caused by exon skipping [160]. Despite the promise shown by Banskota et al. in targeting the retina with VLPs [158], there is still a question whether the photoreceptor outer segment barrier can be crossed. The VLPs display similar properties to enveloped vectors (such as AAVs and lentivirus), except they are designed to deliver protein instead of DNA/RNA. Hence, they are ideal for targeting the RPE since it is a monolayer. Instead, the photoreceptors’ outer segment structure, with numerous flattened membrane discs filled with double phospholipid bilayers, will likely to be more difficult to penetrate.

Besides the non-viral delivery of CRISPR/Cas cargo to the retina, non-viral delivery methods have also been reported, such as delivering the CRISPR components as pure RNPs, as shown by Jang et al. [166]. There is a potential of CRISPR applications with base editing and prime editing in the retina. In this context, RNPs of adenine or cytosine base editors (ABE and CBE, respectively), exhibited different editing patterns than plasmid encoded ABEs/CBEs, with less off-target events due to the short life span of the RNPs, and without sacrificing the on-target ability, as shown by Jang et al. and colleagues [166]. However, this falls outside the scope of this review since the focus is on non-viral approaches for the delivery of the CRISPR/Cas cargo.

A summary of the selected therapeutic application of nanoparticles in the retina is shown in Table 1, including some CRISPR applications.

## 8. Conclusions

In this review, we examined the status of different non-viral delivery platforms of therapeutic reagents, in particular CRISPR/Cas cargo, to the retina to overcome current structural barriers that hinder their efficiency and efficacy. Those barriers have complicated structures and represent a hurdle for the delivery of CRISPR/Cas cargo that is yet to be fully addressed.

CRISPR/Cas systems have empowered researchers and clinicians with an unprecedented toolkit with multiple applications in medicine, ranging from direct genome editing to epigenetics gene regulation and base and prime editing. The history of science will place CRISPR/Cas technologies among the major breakthrough discoveries in therapeutic applications. CRISPR not only expanded our knowledge in the molecular biology of disease mechanisms, it opened up a whole new chapter in therapeutic applications. Due to the space limitations, this review only focuses on the major strategies for the delivery of the CRISPR toolkit. However, several recent reviews discussed its current applications in medicine in more detail [11,25,26,186,187,188,189,190].

One of the major challenges in the current therapeutic era is the delivery of CRISPR tools into living cells in vivo. Due to their low immunogenicity and high efficiency of transduction, AAVs are the most popular therapeutic delivery vehicle, which has been in clinical use for a long time. However, the large molecular size of the CRISPR components creates a major challenge for their packaging into AAV capsids. Current research in the CRISPR field aims to engineer smaller size Cas proteins to overcome the packaging capacity of AAV vectors. Indeed, the emergence of nanotechnology as a potent drug delivery platform opens up new avenues of research and shifts the focus towards non-viral delivery platforms. Nanoparticles have been in clinical use for decades and their potential therapeutic advantages have been widely explored. The potential of CRISPR delivery by nanoparticles is very attractive and more research into the scope of their applications, as well as their limitations, is required. Needless to mention, as the CRISPR technologies are vastly advancing, greater considerations into their ethical applications for in vivo applications should also be discussed [191].

While the in vitro and in vivo applications of non-viral gene delivery have been explored, progress towards clinical applications have been hampered by challenges, including its uptake and stability in target cells. The first FDA-approved CRISPR gene editing in clinical trials was to treat inherited retinal disease using AAV as the delivery vehicle [14]. More research into the stability and efficacy of non-viral delivery platforms in the retina is paramount, particularly with the aim to improve the penetration of the photoreceptor outer segment multiple membrane discs, which is only successfully achievable by AAV vectors so far. Despite the advances in the design of NPs, their number of clinical applications are still below the expected, partially because, as a field, NP design is still under development in the translational gap between animal models and human phases. More understanding of the pathological and physiological properties of the NPs is needed.

In conclusion, CRISPR/Cas systems need to be delivered into the retina using safe and effective non-viral delivery mechanisms. In light of the limited knowledge about the stability, safety, and potency of non-viral delivery platforms for CRISPR in the eye, as of now, AAV vectors remain the primary delivery strategy for CRISPR in the retina, and are likely to remain so until different delivery approaches that can equal their efficiency while overcoming the molecular size limits and increasing the safety and versatility are developed. The focus is now between finding smaller size Cas proteins that can be packaged into AAVs and developing non-viral delivery platforms that are effective and safe.

## Figures and Tables

**Table 1:** Selected examples of nanoparticle cargo delivery in the retina. (* refers to the use of CRISPR).

Delivery Approach/Route	Cargo	Efficiency/Specificity	Application	References
Lipid nanoparticles—Subretinal	mRNA	Expression mainly in RPE, limited expression in Müller glia	In vivo	[167]
Lipid nanoparticles—intravitreal	bevacizamab	Provides 1.5-times higher drug concentration in the vitrous of rabbits	In vivo	[168,169]
Immune nano-liposomes	Antiangiogenic epithelial-derived factor	Significantly reduces CNV formation by binding to normal choroidal vessels	In vivo	[170]
Polymeric nanoparticles	Anti-Flt1 peptide-HA conjugates	Reduces neovascularization and diabetic retinopathy in rats	In vivo	[171,172]
Polymeric nanoparticles—suprachoroidal	VEGF expression plasmid	Reporter gene expression detected in the photoreceptor and RPE layers of rat retina	In vivo	[173]
IgG-gold nanoparticles—subretinal	IgG/protein	Immunolabeling of IgG is seen in the RPE and photoreceptors’ outer segment, 1 week post-injection	In vivo	[174]
DNA-wrapped gold nanoparticles—transfection	DNA	GFP expression in ARPE19 cells—low efficiency	In vitro	[131] *
Poly(lactic-co-glycolic)acid microspheres (PLGA)-Chitosan nanoparticles—intravitreal	K5 plasmid	Area of CNV was reduced 1-week post-injection, and expression of VEGF was downregulated in rat retina	In vivo	[175,176]
PLGA nanospheres—intravitreal	Anti-VEGF aptamer EYE001	Stabilisation of the anti-VEGF aptamer in rabbit sclera for up to 20 days.	In vitro, ex vivo	[177,178,179,180]
Polylactide (PLA) nanoparticles—intravitreal	Rh-6G and Nr-red fluorochromes	Rh staining in neural retina and RPE up to 4 months post-injections	In vivo	[181]
Magnetic nanoparticles—subretinal/intravitreal	Fluorescent magnetic nanoparticles	Potassium blue staining retained in RPE for several day post-injection	In vivo	[148]
Supramolecular Nanoparticles—intravitreal	Dual Cas9/sgRNA-plasmid (RS1 gene)	RS1/GFP successfully incorporated into the mouse retina by CRISPRCas9-mediated knock-in	In vitro, In vivo	[182] *
Carbon nanoparticles—intravitreal	Cas9/sgRNA and HDR of RS1 gene conjugated to nanoparticles	Insertion of RS1 mutation resulted in pathological features of XLRS in iPSCs and mouse retina	In vitro, In vivo	[183] *
Lipid nanoparticles—Subretinal	Liposome-protamine DNA complex (LPD) carries *Rpe65* gene.	LPD promotes cell-specific delivery of LPD into the RPE and long-term expression of *Rpe65*.	In vivo	[184] *
pH-responsive silica-metal-organic nanoparticles (SMOF NP)—subretinal	Cas9-RNP guided of the stop codon in Ai9 locus	SMOF NP achieve efficient editing in Td-Tomato Ai14 mouse.	In vivo	[185] *
